# Mucocèle appendiculaire: à propos d'un cas observé à Lubumbashi

**DOI:** 10.11604/pamj.2014.18.36.2347

**Published:** 2014-05-09

**Authors:** Eric Wakunga, Olivier Mukuku, Marcellin Bugeme, Moïse Tshiband, Audifax Kipili, Pitchou Mobambo, Willy Arung, Warach Wakunga

**Affiliations:** 1 Cliniques Universitaires de Lubumbashi, RD Congo; 2 Polyclinique de l'Amitié de Lubumbashi, RD Congo; 3 Hôpital Jason Sendwe de Lubumbashi, RD Congo

**Keywords:** Mucocèle appendiculaire, Tumeur mucosécrétante appendiculaire, Appendicite, Lubumbashi, Appendiceal mucocoele, Muco-secreting appendiceal tumor, appendicitis, Lubumbashi

## Abstract

La mucocèle appendiculaire est une entité pathologique rare, mais potentiellement dangereuse, elle se présente sous différentes formes cliniques. Nous rapportons ici un cas d'une patiente âgée de 49 ans sans antécédents chirurgicaux chez qui nous avons découvert d'une façon fortuite cette affection. La clinique était celle d'un syndrome appendiculaire aigu patent et elle révélait une masse dans la fosse iliaque droite. Les examens de laboratoire ont montré une hyperleucocytose et une vitesse de sédimentation augmentée. L’échographie a démontré une masse kystique péricaecal. La patiente a subi une appendicectomie avec cæcectomie partielle et la pièce opératoire appendiculaire mesurait 153 mm de longueur et 64 mm de diamètre. L'analyse anatomopathologique de celle-ci a confirmé le diagnostic de mucocèle appendiculaire sans cellules de malignité. Les suites opératoires ont été simples et la patiente est sortie au cinquième jour postopératoire.

## Introduction

Affection rare dans le monde, la mucocèle appendiculaire ou tumeur mucosécrétante appendiculaire est définie comme une distension liquidienne de la lumière appendiculaire par accumulation de mucus. La distension mucineuse de la lumière appendiculaire peut être d'origine tumorale ou non, bénigne ou maligne [1].

Selon la littérature, elle représente 0.15 à 0.6% des pièces d'appendicectomie [2–7]. Cliniquement, elle est asymptomatique dans 25 à 30% des cas et se manifeste par des douleurs chroniques de la fosse iliaque droite dans 70 à 75% des cas [6, 7]. En préopératoire, il est essentiel de reconnaître une mucocèle appendiculaire, pour pouvoir adapter le geste chirurgical. L'imagerie joue actuellement un rôle important dans le diagnostic, mais le diagnostic définitif est basé sur l’étude histologique, qui doit être systématique pour toutes les pièces d'appendicectomie. Son traitement va de la simple appendicectomie dans les formes bénignes, à l'hémicolectomie droite pour cancer dans les mucocèles malignes [8].

La mucocèle appendiculaire pose le double problème de sa malignité éventuelle et du risque de maladie gélatineuse du péritoine (pseudomyxome péritonéal) en cas de perforation. Nous rapportons un cas traité dans le service de Chirurgie des Cliniques Universitaires de Lubumbashi en République Démocratique du Congo. Notre objectif est de montrer les difficultés diagnostiques de cette affection dans notre pratique.

## Patient et observation

Madame N.N âgée de 49 ans a consulté le 24 octobre 2009 pour des douleurs de la fosse iliaque droite de moyenne intensité, sous forme de torsion, sans horaire, non calmées par des antalgiques moyens, irradiant à la fosse iliaque gauche et à la jambe droite évoluant depuis 15 jours. Elle se plaignait également des diarrhées et des vomissements. Elle présentait comme seul antécédent une hypertension artérielle depuis 10 ans stabilisée par du Captopril et des mesures hygiéno-diététiques.

L'examen clinique a mis en évidence, dans la fosse iliaque droite, une forte sensibilité et une masse ferme, sensible, aux contours imprécis et de surface régulière, plongeant dans le pelvis; et un péristaltisme accéléré, au toucher vaginal, une sensibilité et une masse dans le cul-de-sac latéral droit ont été notées.

Sur le plan paraclinique, la numération formule sanguine a montré une hyperleucocytose à 10000 globules blancs par millimètre-cube et une vitesse de sédimentation à 36 millimètres à la première heure. L’échographie abdominale faite a montré une masse hypoéchogène d'allure digestive, hétérogène, indépendante du psoas droit et de la vessie, mesurant 149 mm de long sur 61 millimètres de diamètre, refoulant les annexes droites en arrière. Les éléments cliniques et paracliniques faisaient penser à un processus infectieux aigu de l'appendice, évoquant un plastron appendiculaire.

**Figure 1:**
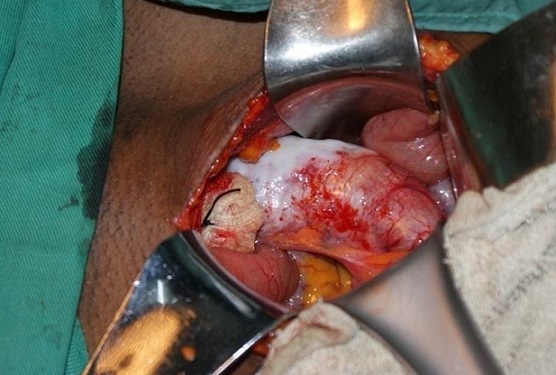
Vue opératoire (avant appendicectomie)

**Figure 2:**
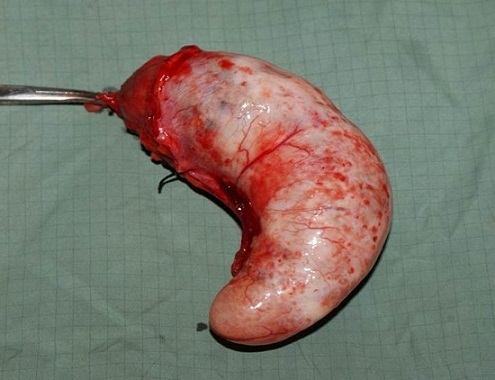
Pièce opératoire (vue de profil)

**Figure 3:**
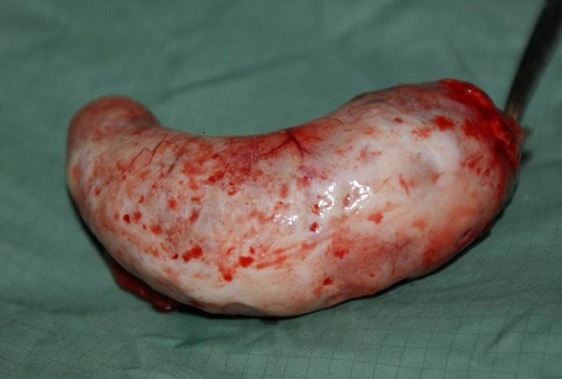
Pièce opératoire (vue de face, Dimensions: 153 mm x 64 mm)

**Figure 4:**
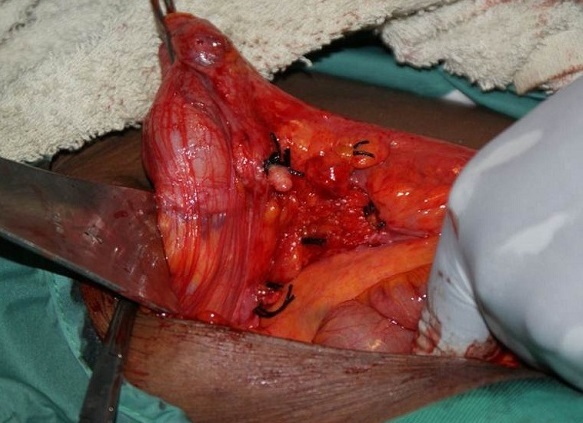
Vue opératoire (après appendicectomie)

Notre patiente a été opérée en urgence: une large incision de Mc Burney a été faite et a permis de découvrir une masse kystique oblongue bien limitée de 15.3 centimètres de longueur et 6,4 centimètres de diamètre avec une base saine (Figure 1, Figure 2, Figure 3) permettant une appendicectomie sans difficulté. Devant l'intégrité de la paroi de la mucocèle appendiculaire et la propreté de la cavité abdominale, nous avons pris la précaution d'exciser le caecum à la base de l'appendice (Figure 4) tout en protégeant la contamination de la cavité péritonéale de toute sécrétion intraluminale.

L'analyse anatomopathologique de la pièce opératoire a confirmé le diagnostic de mucocèle appendiculaire sans cellules de malignité, de type cystadénome mucineux. Les suites opératoires ont été simples et la patiente est sortie au cinquième jour postopératoire. Elle a été revue à six mois puis à un an et ensuite à deux ans; aucun signe patent de récidive locorégionale n'a été notée.

## Discussion

La mucocèle appendiculaire ou tumeur mucosécrétante appendiculaire est définie comme une dilatation kystique de la lumière de l'appendice à la suite d'une accumulation intraluminale de sécrétions mucineuses, translucides, gélatineuses, pouvant toucher soit la totalité de l'organe, soit un segment le plus souvent distal. La distension mucineuse de la lumière appendiculaire peut être d'origine tumorale ou non, bénigne ou maligne [1, 8].

Décrite pour la première fois par Rokitansky en 1842 et nommée par Feren en 1876 [9, 10], la mucocèle appendiculaire est une affection peu fréquente représentant 0.15 à 0.6% des appendicectomies [2–7, 9]. Dans notre milieu, il s'agit d'un cas confirmé de mucocèle appendiculaire sur 874 d'appendicectomies réalisées dans le service sur une période de 5 ans, ce qui donne une fréquence de 0.001%. Sa fréquence serait sous-estimée ici, du fait que certaines pièces d'appendicectomie n'ont pas bénéficié systématiquement d'un examen histologique.

Elle est due à une dilatation de la lumière appendiculaire à la suite d'accumulation de sécrétions mucineuses intraluminales.

Elle touche préférentiellement l'adulte avec un âge moyen entre 50 et 60 ans [4–6, 11, 12] comme nous l'avons noté dans notre observation. Mais la mucocèle appendiculaire peut aussi toucher les enfants; c'est le cas de Duquenoy [13] qui a rapporté cette pathologie chez 5 enfants âgés de 4 à 13 ans souffrant de mucoviscidose. De même, Ekert [14], en 1998 en France, signale une mucocèle appendiculaire chez un enfant âgé de 4,5 ans atteint de la mucoviscidose.

En ce qui concerne le sexe, il s'agissait d'une femme dans notre observation. Le sex-ratio est variable d'une série à l'autre et serait plutôt en faveur d'une prédominance féminine dans les dernières études [4, 5, 9–11]. Par contre, une prédominance masculine a été notée par Souei-Mhiri [6], dans son étude menée entre 1991 et 1998 à Sousse, qui a trouvé 6 hommes contre 4 femmes dans une série de 10 cas.

Sur le plan anatomopathologique, on distingue quatre types de lésions histologiques (par ordre de gravité croissante) [1, 3, 4, 11, 12]:

- Le *kyste rétentionnel* correspondant à l'accumulation de mucus par obstruction de la lumière appendiculaire. En réponse à l'obstruction, la muqueuse devient hyperplasique et hypersécrétante; des changements dégénératifs progressifs surviennent avec apparition de cellules cuboïdes. La paroi appendiculaire devient atrophique et peut être remplacée ultérieurement par du tissu conjonctif.
- L’*hyperplasie villeuse épithéliale*: l'appendice est normal ou légèrement dilaté avec une muqueuse amincie, les lésions sont limitées à la muqueuse et arrangées en structures papillaires fines sans atypies ni mitoses.
- Le *cystadénome mucineux* (tumeur mucineuse bénigne): l'appendice est dilaté par le mucus et la lumière est tapissée par un épithélium mucosécrétant unistratifié. Des formations papillaires peuvent exister, mais l’épithélium est habituellement plat. Certains degrés de dysplasie peuvent être retrouvés associées à des atypies ou des mitoses.
- Le *cystadénocarcinome mucineux* (tumeur maligne) est caractérisé par un haut degré d'atypies cellulaires et de mitoses, un envahissement conjonctif par les cellules néoplasiques et une présence de cellules néoplasiques dans l’épanchement muqueux intra-péritonéal. Sur le plan clinique, la mucocèle appendiculaire peut être totalement asymptomatique (dans 25 à 30% des cas) et serait découverte fortuite au cours d'un examen échographique ou au cours d'une laparotomie pour une autre lésion [4, 6, 7]. Ailleurs, différents tableaux cliniques sont retrouvés [6, 7, 11]:
- Un tableau simulant, dans la majorité de cas, une appendicite aiguë (simple ou compliquée), conduisant en général à une intervention chirurgicale d'emblée. Dans ces formes douloureuses, la douleur peut être isolée et évoluer sous un mode subaigu ou chronique.
- Les formes pseudotumorales: la lésion se présente comme une masse mobile ou fixe de la fosse iliaque droite ou alors comme une abdomino-pelvienne. Ces formes sont plus accessibles à un diagnostic préopératoire à partir d'arguments cliniques et surtout radiologiques.
- Les formes compliquées de volvulus de la masse tumorale, d'infection pouvant aller jusqu’à la suppuration et la perforation ou de rupture dans le péritoine [6]. S'agissant de l'observation que nous rapportons, la clinique évoquait un tableau de plastron appendiculaire. L'imagerie médicale s'appuyant sur l'exploration échographique et scannographique permet le plus souvent d’évoquer son diagnostic et de proposer une prise en charge chirurgicale spécifique. L'examen anatomopathologique de la pièce opératoire est systématique pour poser le diagnostic positif et pour la recherche de signe de malignité [15]. L’échographie met en évidence une masse kystique de la fosse iliaque droite à contenu plus ou moins hypoéchogène donnant l′image dite de la«*peau d′oignon* ». C'est une image de masse, très peu échogène, avec renforcement postérieur que l′on évoque, de nature liquidienne. Cette masse contient des couches échogènes, en arc de cercle et en zone déclive mais non mobiles au changement de position. Cette image permet de proposer le diagnostic préopératoire de mucocèle appendiculaire [4, 12]. Malheureusement, dans notre observation, ceci n'a pas été le cas.

Au scanner, elle apparaît sous forme d'une masse à base caecale, arrondie et bien limitée, à paroi fine, avec fines calcifications pariétales. Sa paroi peut être épaissie, irrégulière, avec nodules prenant le constraste, orientant vers un cystadénocarcinome; cependant, il n'existe pas de signe radiologique permettant d'affirmer ou d'exclure avec certitude la malignité de la tumeur appendiculaire sous-jacente [1, 16].

Faute des moyens financiers, nous n'avons pas pu faire un scanner. D'où l'importance de l'imagerie médicale et des opérateurs patentés pour bien orienter le clinicien afin d'aboutir à un diagnostic précis en préopératoire conditionnant ainsi la prise en charge, car la rupture d'une mucocèle appendiculaire, quel que soit le stade, dans la cavité péritonéale aboutit à un pseudomyxome péritonéal appelé aussi «maladie gélatineuse du péritoine&!187;. Son pronostic varie selon qu'il s'agit d'un adénome ou d'un adénocarcinome mucineux, mais le pseudomyxome péritonéal reste une affection grave. C'est la raison pour laquelle l'exérèse d'une mucocèle appendiculaire lors d'une appendicectomie, doit se faire absolument sans effraction de sa paroi [16].

## Conclusion

Devant la découverte ou la persistance d'une masse de la fosse iliaque droite ou un syndrome appendiculaire, la mucocèle appendiculaire mérite d’être évoquée bien qu'elle soit rare. Le diagnostic préopératoire est possible et important nécessitant ainsi la réalisation d'une échographie et/ou d'un scanner car il permet d'alerter le chirurgien sur le risque de rupture pendant la chirurgie et d’éviter un pseudomyxome du péritoine. L’étude histologique systématique de toute pièce d'appendicectomie doit être de règle. La prise en charge consiste en une l'appendicectomie simple dans la majorité des cas, mais une hémicolectomie droite doit être faite systématiquement dans les cas où les signes de malignité locale sont patents ou confirmés par l'analyse anatomopathologique de la pièce opératoire.
